# A critical analysis of the current TNM classification for differentiated thyroid carcinoma in young patients: Time for a change?

**DOI:** 10.3389/fendo.2022.939131

**Published:** 2022-10-19

**Authors:** Wenlong Wang, Ning Bai, Xinying Li

**Affiliations:** ^1^ Thyroid Surgery Department, Xiangya Hospital, Central South University, Changsha, China; ^2^ National Clinical Research Center for Geriatric Disorders, Xiangya Hospital, Central South University, Changsha, China

**Keywords:** differentiated thyroid carcinoma (DTC), cancer-specific survival (CSS), TNM, prognosis, C-index

## Abstract

**Background:**

The current TNM classification that simply classifies differentiated thyroid carcinoma (DTC) patients younger than 55 years into stage I and stage II based on the presence or absence of distant metastases has been questioned. In this study, we reexamined the impact of T status and N status on prognosis and then developed a new prediction model to improve the predictability of cancer-specific survival (CSS) in young patients.

**Materials and methods:**

Kaplan–Meier method was applied to calculate the CSS. Multivariable Cox proportional hazards models were used to assess the impact of T status and N status on CSS after adjustment for known covariates. The area under the receiver operating characteristic curve (AUC), C-index, Bayesian information criterion (BIC), and Akaike information criterion (AIC) were calculated to compare model performance.

**Results:**

A total of 9,242 DTC patients younger than 55 years were enrolled in the study. After adjusting for gender, age at diagnosis, race, pathology subtype, N stage, and M stage, T3 disease [hazard ratio (HR): 3.78, *P* = 0.006] and T4 disease (HR: 7.96, *P <* 0.001) remain independent predictors of CSS. Similarly, the 10-year CSS rate of N1b disease (HR: 3.78, *P <* 0.001) was significantly higher than that of N0 disease after adjustment. Moreover, Kaplan–Meier survival analysis showed that the 10-year CSS of stage II disease in younger patients with DTC showed a sharp decrease compared with that in older patients with DTC (74.47% vs. 98.43%, *P* < 0.001). Furthermore, a modified TNM staging system based on significantly prognostic T stage and N stage was established, which showed better performance than the current TNM staging system (*P* < 0.05). The new prediction model is also applicable to papillary thyroid carcinoma patients and follicular thyroid carcinoma patients.

**Conclusions:**

This is the first study to question the rationality of the current TNM staging system for patients younger than 55 years and successfully develop a new prognostic model, which improves prognostic stratification and guides individualized management.

## Introduction

In the past decades, the prevalence of thyroid carcinoma (TC) has increased rapidly worldwide (1, 2). Differentiated TC (DTC), including papillary TC (PTC) and follicular TC (FTC), is the most common histological subtype with a favorable prognosis (3). Yet, 15%–20% of DTC patients suffer either distant metastasis or recurrence after initial treatment, a small number of patients even die of DTC (4, 5). Therefore, it is vital to identify high-risk patients early at diagnosis to adopt more aggressive treatment.

Currently, multiple classification systems have been created to inform DTC clinical management, and the eighth edition of the TNM staging system (TNM-8) is the most widely used to predict cancer progression and overall mortality (6). Unlike most other carcinomas, patient’s age at diagnosis has been recognized as a critical factor in the prognostic evaluation of DTCs (7, 8), which is incorporated into the TNM staging system. This non-anatomic factor combined with anatomic extent to stage DTC has been used since 1983 (9). Patients with DTC younger than 55 years are divided into stage I (without distant metastatic disease) and stage II (with distant metastatic disease), whereas patients with DTC aged 55 years or older can be classified into stages I–IVB (10). However, many experts questioned and are concerned with its usefulness in clinical staging and prognostic validity (11–15), especially for younger patients (<55 years) (16). Pathak et al. (17) showed that non-metastatic stage II DTC and metastatic stage II DTC were significantly different in terms of disease-free survival and cancer-specific survival (CSS), which proved that younger patients with metastatic DTC were understaged and that stage II DTC could not be uniformly considered as a low-risk disease. Moreover, a recent study based on two larger cohorts demonstrated that lymph node involvement was associated with compromised survival in young patients (18). Also, our previous study found that a large nodule size significantly increased the risk of unfavorable events in younger patients (19). The abovementioned results suggest that it is inappropriate to classify patients younger than 55 years without metastatic disease into uniform stage, and that stage II DTC in older patients and stage II DTC in younger patients are not equal in overall survival. Therefore, there is considerable room for improvement in the current TNM staging system.

In an effort to design a reasonable clinical staging system for young patients, we sought to perform a detailed analysis of the impact of different T stages and N stages on CSS using a large DTC cohort. Specifically, we compared the CSS in American Joint Committee on Cancer (AJCC)-TNM stage II DTC in younger and older patients. Furthermore, a new modified TNM staging system was established to verify the validity of staging DTC in young patients, including FTC patients and PTC patients. This is, to our knowledge, the first study to present a new risk stratification that accurately reflects the outcomes for DTC.

## Materials and methods

### Study population

All data were acquired from the Surveillance, Epidemiology, and End Results (SEER) database (https://seer.cancer.gov) that was released in April 2021. The SEER database was the largest public cancer database that represents 27.8% of the US population (20). We extracted data of patients diagnosed as having DTC [International Classification of Diseases for Oncology (ICD-O)-3 codes 8450–8460, 8350, 8340–8344, 8260, 8050, 8330–8335, and 8290] from the SEER 21 registry during the period 2000–2018. Collected patient data included sex, age at diagnosis, histology, ethnicity, and T/N/M stage. Survival information was also extracted, including survival status, the time to last follow-up, and cause of death. Patients were selected based on the following inclusion criteria: 1) histologically confirmed DTC; 2) clearly denoted T/N/M stage; 3) performed thyroidectomy with neck lymph node dissection; 4) the follow-up period was over 10 years. Patients with unknown or missing clinicopathologic data were excluded. In total, 9,242 patients with DTC were found eligible for the study.

### Definition

Younger patients (<55 years old) diagnosed as having DTC had been classified into either stage I (without distant metastatic disease) or stage II (with distant metastatic disease), and stage II DTC contained T2N1M0, T1N1M0, and T3a/T3bNanyM0 in patients aged 55 years or older based on TNM-8. Briefly, N status was simply classified into three groups (N0, N1a, and N1b), and T stages were subdivided into four groups (T1, T2, T3, and T4) for better prediction of the prognosis. The endpoint of this study was CSS, which was defined as the time interval from the initial thyroidectomy to the last observation date or death caused by DTC, as described previously (21).

### Ethics approval

All methods were performed in accordance with the relevant regulations and guidelines. All data were accessed from the public database and did not involve any non-human subjects in accordance with the Office for Human Research Protection. Our research was waived from review by the ethics committee of Xiangya Hospital of Central South University.

### Statistical analysis

Continuous variables are expressed as means with standard deviations (SDs) or medians with range, and categorical variables are displayed as numbers with percentages. Kaplan–Meier method was applied to draw survival curves for CSS, and the log-rank test was used to determine significance. The impacts of T stages and N stages on CSS were assessed by the Cox proportional hazards regression analysis after adjustment for known covariates. Ninety-five percent confidence intervals (CIs) and hazard ratios (HRs) were estimated. The area under the receiver operating characteristic curve (AUC) and C-index were calculated to determine the discrimination ability. Two-sided *P* < 0.05 was considered to be statistically significant. Statistical analyses were performed using SPSS version 22.0 (IBM Corp., USA), and pictures were drawn by GraphPad Prism version 8.0 (GraphPad Software Inc.).

## Results

### Baseline characteristics

A total of 9,242 DTC patients were enrolled in the study, and patient characteristics were shown in **Table 1**. In this study, 2,059 (22.3%) were men and 7,183 (77.7%) were women. The median age at diagnosis was 41 years. Patients were predominantly white (79.7%) and had PTC (89.6%). The distribution of N classification was 1,037 (11.2%) with N1b stage, 1,550 (16.8%) with N1a stage, and 6,655 (72.05) with N0 stage. The proportions of patients with T stage of I, II, III, and IV were 31.8% (n = 2,938), 33.1% (n = 3,055), 30.9% (n = 2,853), and 4.3% (n = 396), respectively. According to the TNM-8, 9,148 (99.0%) and 94 (1.0%) patients were classified as stage I and stage II, respectively. During the median follow-up of 110.2 months, there were 94 (1.02%) DTC-related deaths.

**Table 1:** The clinical characteristics of differential thyroid cancer patients.

Patient characteristics	n (%) or mean ± SD
Age, years	39.77 ± 9.94
Gender	
Male Female	2,059 (22.3) 7,183 (77.7)
Ethnicity	
Black White Other	692 (7.5) 7,369 (79.7) 1,181 (12.8)
Pathology subtype	
PTC FTC	8,283 (89.6) 959 (10.4)
T stage	
T1 T2 T3 T4	2,938 (31.8) 3,055 (33.1) 2,853 (30.9) 396 (4.3)
N stage	
N0 N1a N1b	6,655 (72.0) 1,550 (16.8) 1,037 (11.2)
M stage	
M0 M1	9,148 (99.0) 94 (1.0)
8th edition TNM stage	
I II	9,148 (99.0) 94 (1.0)
Follow-up period (months), median (IQR)	110.2 (1-179)
Cancer-specific mortality	94 (1.02)

SD, standard deviation; PTC, papillary thyroid carcinoma; FTC, follicular thyroid carcinoma; IQR, interquartile range.

### Contradiction with the current TNM classification for younger patients

The rate of 10-year CSS was 99.83% (2,935/2,938), 99.67% (3,045/3,055), 98.21% (2,802/2,853), and 92.39% (340/368) for T1, T2, T3, and T4 disease, respectively. Compared with T1 disease, the crude HR of CSS was 1.59 (95% CI 0.54–4.66, *P* = 0.4), 8.85 (95% CI 3.52–22.24, *P <* 0.001), and 35.84 (95% CI 13.78–93.21, *P <* 0.001) for T2, T3, and T4 disease, respectively. After adjusting for gender, age at diagnosis, race, pathology subtype, N stage, and M stage, the adjusted HRs of T3 (95% CI 2.15–6.23, *P* = 0.006) and T4 (95% CI 2.82–22.46, *P <* 0.001) disease remained significant. Similarly, the presence of N1b disease (95% CI 2.15–6.23, *P <* 0.001) was an independent predictor of worse CSS after adjustment (**Table 2**). These results suggest that it is inappropriate to divide patients younger than 55 years without metastatic disease into uniform stage I regardless of T and N stages.

**Table 2:** T classification and N classification impact on 10-year cancer-specific survival.

Variables		Unadjusted		Adjusted^a^	
		HR (95% CI)	*P*	HR (95% CI)	*P*
T stage	T1	Reference		Reference	
	T2	1.59 (0.54-4.66)	0.40	0.94 (0.32-2.81)	0.92
	T3	8.85 (3.52-22.24)	<0.001	3.78 (2.15-6.23)	0.006
	T4	35.84 (13.78-93.21)	<0.001	7.96 (2.82-22.46)	<0.001
N stage	N0	Reference		Reference	
	N1a	1.14 (0.59-2.22)	0.69	1.35 (0.65-2.80)	0.43
	N1b	6.64 (4.32-10.22)	<0.001	3.78 (2.15-6.23)	<0.001

HR, hazard ratio; CI, confidence interval.

^a^Adjusted for age, race, sex, pathology subtype, M stage, N stage, or T stage.

**Figure 1:**
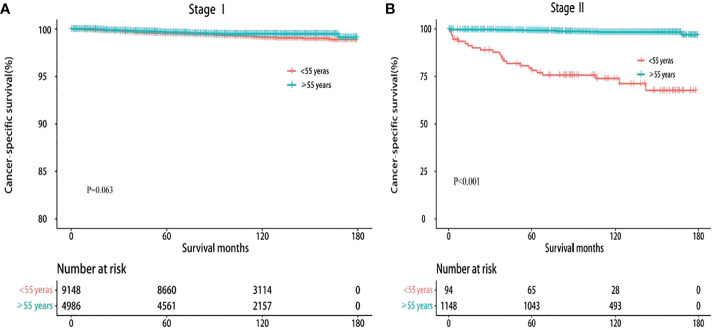
Kaplan–Meier survival curves of cancer-specific survival based on the current TNM-8. **(A)** Stage I. **(B)** Stage II.

Furthermore, prior studies questioned the rationality of classifying both younger people with metastasis and older patients without distant metastasis into stage II under current AJCC guidelines (16, 22). Kaplan–Meier survival analysis showed that the 10-year CSS of stage II disease in younger patients with DTC showed a sharp decrease compared with that in older patients (74.47% vs. 98.43%, *P* < 0.001). However, there was no statistically significant difference in stage I between younger and older patients (99.23% vs. 99.49%, *P* = 0.063) (**Figure 1**). These results confirm that all stage II DTCs cannot be uniformly considered as a low-risk disease.

### Constructing the modified TNM staging system for young patients

**Figure 2:**
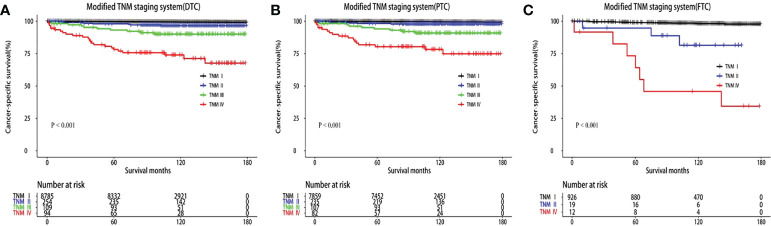
Kaplan–Meier survival curves of cancer-specific survival according to the modified TNM staging system. **(A)** Differentiated thyroid carcinoma (DTC). **(B)** Papillary thyroid carcinoma (PTC). **(C)** Follicular thyroid carcinoma (FTC).

**Figure 3:**
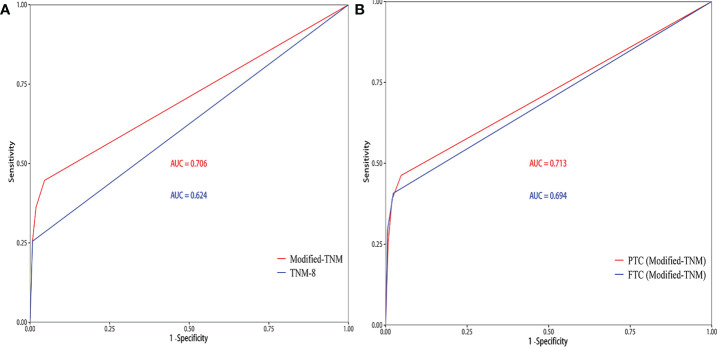
The area under the receiver operating characteristic curve (AUC) of the prediction model. **(A)** TNM-8 and the modified TNM staging system for patients younger than 55 years. **(B)** Performance of the modified TNM staging system when separately applied to papillary thyroid carcinoma (PTC) patients and follicular thyroid carcinoma (FTC) patients.

T3, T4, and N1b stages were found to act as independent predictors of CSS for patients younger than 55 years. A modified TNM staging system based on significantly prognostic T stage and N stage was established to predict CSS (**Table 3**), as follows: modified TNM stage I (T1–2NanyM0 and T3N0-1aM0), modified TNM stage II (T3N1bM0 and T4N0-1aM0), modified TNM stage III (T4N1bM0), modified TNM stage IV (TanyNanyM1). The 10-year CSS rates were 99.55%, 97.04%, 90.9%, and 74.5%, respectively (*P* < 0.001) (**Figure 2A**). The AUC of the modified TNM staging system was significantly higher than that of TNM-8 (0.706 vs. 0.624, *P* < 0.05) (**Figure 3A**). Comparing TNM-8 and the modified TNM staging system, the C-index was 0.716 and 0.628, the Akaike information criterion (AIC) was 1,510.64 and 1,513.18, and the Bayesian information criterion (BIC) was 1,554.92 and 1,557.46, respectively. These results indicate that the modified TNM staging system shows better prediction.

**Table 3:** Proposal of new modified TNM staging system for younger DTC patients.

Classification	CSS	New TNM	CSS	8th TNM stage
T1-2, N0-1a, M0	99.9%	TNM I	99.55%	TNM I
T1-2, N1b, M0	98.4%			
T3, N0-1a, M0	98.9%			
T3, N1b, M0	97.1%	TNM II	97.04%	
T4, N0-1a, M0	96.9%			
T4, N1b, M0	90.8%	TNM III	90.9%	TNM II
Any T, any N, M1	74.5%	TNM IV	74.5%	

CSS, cancer-specific survival; DTC, differentiated thyroid carcinoma.

### Performance of the modified TNM staging system in predicting cancer-specific survival

In the modified TNM staging system of DTCs, the adjusted HRs increased as the stage increased (HR 5.55, 16.40, and 48.93 for stage II, III, and IV, respectively). Similar results were revealed in PTC and FTC patients (**Table 4** **;** **Figures 2B, C** **)**. The AUC and C-index of the modified TNM staging system for PTC and FTC were 0.713 vs. 0.694 and 0.725 vs. 0.697, respectively (**Figure 3B**), which demonstrated that the modified TNM staging system performed well when separately applied to PTC and FTC.

**Table 4:** Hazard ratios of the modified TNM staging system for cancer-specific survival.

Pathology subtype	Modified TNM stage	Unadjusted		Adjusted^b^	
				HR (95% CI)	*P*	HR (95% CI)	*P*
DTC	TNM I	Reference		Reference	
	TNM II	4.93 (2.34-10.40)	<0.001	5.55 (2.63-11.72)	<0.001
	TNM III	15.68 (7.96-30.86)	<0.001	16.40 (8.16-32.99)	<0.001
	TNM IV	52.23 (32.18-84.78)	<0.001	48.93 (30.01-79.76)	<0.001
PTC	TNM I	Reference		Reference	
	TNM II	4.25 (1.67-10.86)	0.002	4.43 (1.73-11.32)	0.002
	TNM III	18.12 (8.74-37.76)	<0.001	18.48 (8.72-39.18)	<0.001
	TNM IV	53.84 (30.22-95.93)	<0.001	64.63 (35.91-116.34)	<0.001
FTC	TNM I	Reference		Reference	
	TNM II	10.75 (3.13-36.91)	<0.001	9.27 (2.68-32.10)	<0.001
	TNM III	–	–	–	–
	TNM IV	47.34 (19.40-115.53)	<0.001	40.80 (16.41-101.41)	<0.001

HR, hazard ratio; CI, confidence interval; DTC, differentiated thyroid carcinoma; PTC, papillary thyroid carcinoma; FTC, follicular thyroid carcinoma.

^b^Adjusted for age, race, sex, and pathology subtype.

## Discussion

The clinical risk stratification is the cornerstone and essence of individualized DTC management, which provides a guideline in accurately choosing treatment and reflecting the outcomes of DTC. In this regard, the AJCC-TNM staging system was considered to be the gold standard for predicting the mortality risk in cancer patients (23, 24). This is, to our knowledge, the largest retrospective cohort study with longer follow-up times that specifically addresses the proposal of a new risk stratification for DTC patients under 55 years so far and found that it is inappropriate to classify patients younger than 55 years without metastatic disease into uniform stage I, and that stage II DTC in older patients and stage II DTC in younger patients are not equal in overall mortality. These results update our current knowledge of DTC stratification in young patients.

Recently, the revised TNM-8 weakened the impact of lymph node metastasis on prognosis and eliminated the difference between N1b and N1a disease (25). However, increasing evidence demonstrated that N1b disease had significantly worse survival than N1a disease, and there is growing concern that the recurrence and mortality risk are underestimated in patients with N1b disease (26, 27). Cervical lymph node metastases are well-known risk factors for poorer outcomes in patients with PTC, especially for older patients. However, its significance in younger patients has not been well defined. Patients younger than 55 years are usually perceived to have low-risk disease. Zaydfudim et al. (28) showed that lymph node involvement negatively impacts survival in patients with FTC instead of PTC. In an analysis of 49,240 patients with DTC from the SEER registry data, Tran et al. (16) proved that lymph node disease was associated with increased mortality in patients younger than 45 years. Nobuaki et al. (29) found that lymph node metastases had no effect on survival. Of particular interest, some studies (30, 31) suggested that nodal metastasis significantly increased the risk of recurrence albeit with little effect on overall survival. Unfortunately, the above study did not exclude the confounding variables, such as tumor size, presence of distant metastasis, and treatment factors. In the current study, after adjusting for gender, age at diagnosis, race, pathology subtype, T stage, and M stage, N1b disease remains an independent predictor of worse CSS in younger patients. These results indicate that the existing TNM staging system needs to consider the impact of N1b disease on CSS rather than simply classifying patients younger than 55 years without metastatic disease into uniform stage.

Moreover, microscopic extrathyroidal extension (ETE) was deleted from the definition of T3 disease in the recently updated TNM-8, which led to a large number of DTC patients being downgraded in stages (25). The degree of ETE is an important index to determine the assignment of T status, which affects the TNM stage and the choice of patient treatment strategies (32). Gross ETE was recognized as a variable that negatively affects prognosis in DTC, and whether microscopic ETE significantly increases the risk of recurrence and overall mortality remains controversial (33–35). A recent study has reported that microscopic ETE was an independent predictor of recurrence-free survival and significantly associated with poor survival (36). Another study reached the opposite conclusion that microscopic ETE had no effect on the risk of death or recurrence (37). These inconsistent conclusions are due to different design methods, sample sizes, and population-specific institutional datasets. Our study first described the influence of T stage on prognosis in young patients and then revealed that advanced T stage was an independent prognostic factor for CSS. These results indicated that young age was not enough to offset the impact of gross ETE on CSS, so it is clear that the existing TNM-8 overestimates the protective effect of young age.

Based on the shortcomings of the current TNM-8, we established a new modified TNM staging system for DTC in young patients to improve the predictability of CSS with long-term follow-up. Prior studies attempt to incorporate some prognostic genetic markers such as BRAF V600E and TERT promoter mutations into TNM-8 to strengthen its predictability (13, 38). However, it is difficult to apply in real clinics worldwide. In this study, the HRs increased as the stage increased (HR 4.93, 15.68, and 52.23 for stage II, III, and IV, respectively). The modified TNM staging system has significantly superior risk predictability for CSS than the TNM-8 with a C-index of 0.716 vs. 0.628 and an AUC of 0.706 vs. 0.624. Furthermore, a multicenter cohort study indicated that the TNM-8 improved the predictability of CSS in patients with PTC but not FTC (39). FTC is of interest due to its biological behavior being completely different from PTC, which tends to show distant metastasis and vascular invasion at initial presentation (40). Thus, the current AJCC staging guidelines need to be aware of the impact of these differences on prognosis. In our study, the modified TNM staging system was also applicable to PTC patients and FTC patients with good predictive performance.

This study has several limitations. Due to its retrospective design, selection bias was inevitable. In addition, the therapeutic management, including the extent of thyroidectomy, indication of radioactive iodine (RAI) adjuvant therapy, and thyroid-stimulating hormone (TSH) inhibition level, varies from current guidelines. Further studies from larger prospective and multicenter cohorts are necessary to exclude potential bias and validate the generalizability of the modified TNM staging system in young patients. Another limitation is the inherent flaw of the SEER dataset. Some important clinicopathological information such as vascular invasion, family history, and BRAF and TERT promoter mutations were not included because we do not have access to these data. Nevertheless, our findings are based on a large population, and our study used strict inclusion/exclusion criteria, presenting an accurate and reliable conclusion, which might be considered for inclusion in a subsequent AJCC-TNM edition.

## Conclusion

In summary, this is the first study to question the rationality of the current TNM-8 for patients younger than 55 years and present a new prognostic model for patients with DTC, which improved the risk stratification and allowed clinicians to make more individualized treatment strategies.

## Data availability statement

The original contributions presented in the study are included in the article/**Supplementary Material**. Further inquiries can be directed to the corresponding authors.

## Author contributions

WW and NB: Conceptualization and methodology. WW and XL: Formal analysis and data curation. WW and NB: writing—original draft preparation. All authors have read and agreed to the published version of the manuscript.

## Conflict of interest

The authors declare that the research was conducted in the absence of any commercial or financial relationships that could be construed as a potential conflict of interest.

## Publisher’s note

All claims expressed in this article are solely those of the authors and do not necessarily represent those of their affiliated organizations, or those of the publisher, the editors and the reviewers. Any product that may be evaluated in this article, or claim that may be made by its manufacturer, is not guaranteed or endorsed by the publisher.
